# Caregiving Through Another Lens: The History of Long-Term Care

**DOI:** 10.1093/geroni/igaf122.931

**Published:** 2025-12-31

**Authors:** Kathleen Wilber

**Affiliations:** University of Southern California, Los Angeles, California, United States

## Abstract

Long-term care has been a key focus of gerontological research for decades. Passage of the Medicaid program in 1965 led to a boom in the nursing home industry. In 1971 the White House Conference on Aging called for the development of non-institutional services. States launched demonstration programs in home and community- based services (e.g., adult day health care, case management) and seminal research (Weissart,1979; the Channeling Demonstration) assessed “nursing home alternatives.” Although studies failed to demonstrate diversion of high-risk people from placement, policymakers, undeterred, began to enact Medicaid waivers to “rebalance” services for aging in place. Efforts also focused on improving care in nursing facilities. The 1987 Nursing Home Reform Act sought to provide systematic data, expand monitoring, and require input from residents and families. Private sector investments in Assisted Living, offered more home-like environments for those with means. Oversight and litigation expanded. In the early 2000s, culture change programs sought to overhaul how care is provided. This presentation will offer lessons from over five decades of program and policy development. The long-term care landscape includes innovative models and pioneering programs. Yet scandals continue; the COVID pandemic revealed heroes, villains, and examples of horrifying care, facility ownership and hence responsibility is often hidden. On the “alternatives” side, those seeking home and community-based services may find that they are not eligible. Those that are eligible may encounter long waiting lists. What are the lessons learned, where are we, how can we do better?

